# Total Cholesterol Variability and the Risk of Osteoporotic Fractures: A Nationwide Population-Based Cohort Study

**DOI:** 10.3390/jpm13030509

**Published:** 2023-03-11

**Authors:** Dongyeop Kim, Jee Hyun Kim, Tae-Jin Song

**Affiliations:** Department of Neurology, Seoul Hospital, College of Medicine, Ewha Womans University, Seoul 07804, Republic of Korea

**Keywords:** cholesterol, variability, osteoporosis, fracture, hip fracture, vertebral fracture

## Abstract

Several risk factors for osteoporotic fractures have been identified but reports of the association of lipid parameters with the occurrence of osteoporotic fractures have been limited. We aimed to examine whether serum total cholesterol (TC) variability is associated with osteoporotic fractures. The study included 3,00,326 subjects who had undergone three or more health examinations between 2003 and 2008. The primary endpoint was the incidence of osteoporotic fractures, including vertebral, hip, distal radius, and humerus fractures. TC variability was evaluated based on the following three parameters: coefficient of variation (CV), standard deviation (SD), and variability independent of the mean (VIM). A total of 29,044 osteoporotic fracture events (9.67%) were identified during a median of 11.6 years of follow-up. The risk of osteoporotic fractures in the highest quartile was significantly higher compared with the lowest quartile according to the three indices of TC variability with adjusted hazard ratios (HR) and 95% confidence intervals (CI) as follows: CV (HR 1.11, 95% CI [1.08–1.15]), SD (HR 1.07, 95% CI [1.04–1.11]) and VIM (HR 1.07, 95% CI [1.04–1.11]). The Kaplan–Meier curves showed a significantly positive relationship between the higher quartile of TC variability and overall osteoporotic fractures. The association remained significant in subgroup analyses of vertebral and hip fractures, regardless of the indices of TC variability. Our study showed that visit-to-visit TC variability was found to be associated with osteoporotic fracture risk. Maintaining TC levels stable may help attenuate the osteoporotic fracture risk in the future.

## 1. Introduction

Osteoporotic fractures are major complications caused by osteoporosis, resulting in medical and social burdens. The common sites for osteoporotic fractures are the spine, hip, and forearm. However, almost all types of fractures, including the humerus, rib, and pelvis, are increased in patients with low bone density, considered to be due to osteoporosis [1,2]. It has been reported that the lifetime risk of any type of osteoporotic fracture is greater than 40% for women and 13% for men [1], and 9.5% of deaths were listed as being directly caused by fractures [3]. In particular, spine and hip fractures are directly linked to increased mortality and morbidity, with the long-term mortality of patients with these types of fractures being higher than that of the general population due to accompanying comorbidities [3,4,5].

Various clinical risk factors known to be associated with fractures were identified, including female sex, older age, premature menopause, low bone mineral density (BMD), previous fracture, glucocorticoid therapy, and low body weight [6]. Besides, a serum lipid profile has been postulated to be associated with osteoporosis and osteoporotic fractures. Previous meta-analyses demonstrating that taking lipid-lowering agents was associated with increased BMD at various sites, and a lower risk of osteoporotic fractures, also support the association between serum lipid and osteoporosis [7,8,9]. However, cholesterol measurement at a single time point may not be sufficient to reflect the lipid profile of the subject. The variability of cholesterol has been recognized as biologically variable with an average variation of 6.5% [10]. Moreover, in several studies that collected cholesterol levels at a single time point, the relationship between serum lipids and osteoporotic fractures was inconsistent. Therefore, we decided to use cholesterol variability as an alternative measure to the absolute value of cholesterol levels.

Intraindividual variability of several biological measures, including blood pressure, heart rate, and glucose, could be a predictive biomarker of adverse health outcomes [11]. Visit-to-visit cholesterol variability has been found to act as a risk factor for cardiovascular diseases, stroke, and mortality regardless of the cholesterol target levels achieved to lower the risk [12,13,14,15,16]. Moreover, cholesterol variability has been found to be related to a risk of end-stage renal disease, lower cognitive performance, and even Parkinson’s disease [17,18,19]. Based on the fact that bone and vascular tissue have molecular and cellular characteristics in common [20], and cholesterol variability has been suggested to cause dysfunction of the vascular wall and endothelium [14], we hypothesized that vascular dysfunction induced by cholesterol variability may exacerbate bone loss and increase the risk of fractures. In this study, which is based on a national population cohort, involving a large sample size representative of the general population, we aimed to evaluate the role of TC variability in assessing the risk of osteoporotic fractures.

## 2. Methods

### 2.1. Dataset

The National Health Insurance system (NHIS) provides a nationwide cohort that includes eligibility (e.g., age, sex, and socioeconomic variables), medical treatment (according to the medical bills submitted by healthcare providers), a health examination (test results and lifestyle questionnaires), and a medical care institution database (types of medical care institutions, location, equipment and the number of physicians) [21,22,23]. The NHIS was founded in 2000 as a single medical insurer by the Korean government and covers approximately 97% of the South Korean people. Enrollees aged between 40 and 79 years are recommended to receive free health screening examinations every year, and the blood tests are performed after overnight fasting, at least for 8 h, which are conducted only at laboratories that have been accredited and certified by the national institution.

### 2.2. Study Population

**Figure 1:**
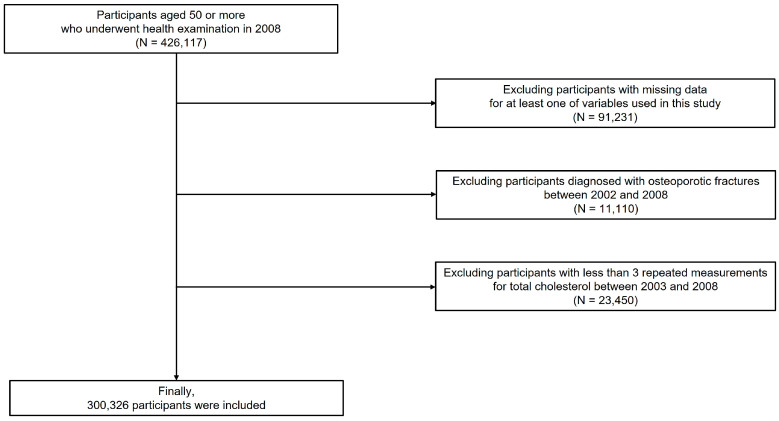
Flowchart of study subjects.

Stratified random sampling was applied to make sure that the selected samples represent the entire population. A total of 426,117 subjects who underwent health examinations in 2008 and were aged 50 years or more were enrolled. Then, those with at least one missing value in demographic or laboratory data (*n* = 91,231), those who had osteoporotic fractures between 2002 and 2008 (*n* = 11,110), and those who underwent cholesterol tests less than three times (*n* = 23,450) were excluded. Finally, 300,326 subjects were included in this study (Figure 1). Due to the retrospective analysis of the NHIS data, which does not contain personally identifiable information, informed consent was waived. The Institutional Review Board of the Ewha Womans University Seoul Hospital approved this research (SEUMC 2022-07-053).

### 2.3. Assessment of Total Cholesterol Variability

TC variability was determined as the variation in three or more TC values identified between 2003 and 2008. Three different measures of variability are presented in this study: standard deviation (SD), coefficient of variation (CV = SD/mean), and variability independent of the mean (VIM = 100 × SD/mean^β^, where the regression coefficient β is determined by taking the natural logarithm of the SD and dividing it by the natural logarithm of the mean). The smaller these values are, the closer the distribution is to the average value, which means low variability. The number of TC measurements were as follows: three measurements (*n* = 61,951, 52.7%), four measurements (*n* = 15,073, 12.8%), five measurements (*n* = 13,549, 11.5%), and six measurements (*n* = 27,100, 23.0%). 

### 2.4. Study Endpoints

The primary endpoint was the incidence of osteoporotic fractures during the follow-up, based on the ICD-10 codes: vertebral fracture (S22.0, S22.1, S32.0; S32.7, T08, M48.4, M48.5, M49.5); hip fracture (S72.0, S72.1); distal radius fracture (S52.5, S52.6); or humerus fracture (S42.2, S42.3). The Korean Society for Bone and Mineral Research proposed and confirmed the operational definitions of osteoporotic fractures by utilizing NHIS data, with the help of ICD-10 codes and procedure codes [24]. The observation period was determined as the period from enrollment (the first health examination date) to the earliest occurrence of osteoporotic fractures, death, or the end of the study (31 December 2020).

### 2.5. Variables

The date of enrollment in the health examination was designated as the index date for the study. On the index date, the following baseline clinical characteristics were obtained: age, gender, body mass index (BMI), and socioeconomic status. Lifestyle habits including smoking (never, former, and current smoker), drinking alcohol (days per week), and physical activity (days per week) were collected via self-reported questionnaires. Comorbidities, including hypertension, diabetes mellitus, dyslipidemia, stroke, atrial fibrillation, renal disease, and cancer, were defined based on the International Classification of Diseases, Tenth Revision (ICD-10) codes, medication use, and test results from health examination through data between 2002 and the index date. The detailed definitions of comorbidities were demonstrated in supplementary methods and previous studies [25,26,27,28].

### 2.6. Statistical Analysis

Descriptive statistics were utilized to present the results, with mean ± SD being used for continuous variables and numbers (%) for categorical variables. Subjects were categorized into quartiles according to TC variability. An analysis of variance (ANOVA) was conducted to compare continuous variables, while chi-square tests were used for categorical variables. The event rate of the osteoporotic fractures was calculated as the number of events divided by person-years. Kaplan–Meier survival curves were performed to estimate the occurrence of osteoporotic fractures stratified by quartiles of TC variability according to CV and compared using the log-rank test. The Cox proportional hazards model was used to present hazard ratios (HRs) with 95% confidence interval (CI) values to estimate the risk of osteoporotic fractures depending on TC variability. The following potential confounders were adjusted for multivariable analysis: age, sex, BMI, income levels, smoking habits, drinking alcohol, regular exercise, taking lipid-lowering agents, and comorbidities (hypertension, diabetes mellitus, dyslipidemia, stroke, atrial fibrillation, renal disease, and cancer). Further analyses were performed with each fracture site as the outcome. The mean cholesterol level was also adjusted in the multivariable model for sensitivity analysis. In addition, landmark analysis was performed to control immortal time bias, and participants who had osteoporotic fractures within one year after study enrollment were excluded. Statistical analyses were performed using SAS version 9.2 (SAS Institute, Cary, NC, USA), and a significance level of less than 0.05 for the two-sided *p*-value was considered statistically significant.

## 3. Results

### 3.1. Demographics and Clinical Characteristics

The mean age of subjects was 56.3 ± 5.9 years, and 73.7% of them were men. Osteoporotic fractures, including multiple fractures, were identified in 29,044 subjects (9.67%): 15,920 subjects with vertebral fractures (5.30%); 2672 subjects with hip fractures (0.89%); 11,475 subjects with distal radius fractures (3.82%); and 1685 subjects with humerus fractures (0.56%). The baseline characteristics of 300,326 subjects subgrouped by quartiles of TC variability based on the CV are shown in Table 1. Subjects in the highest quartile were older, predominantly female, had a higher BMI, lower socioeconomic status, and more comorbidities, and more subjects were taking lipid-lowering agents than those in the lowest quartile. Risk factors for osteoporotic fractures were identified in this study, which are similar to those already known, including advancing age, female sex, current smoking, frequent drinking, and comorbidities (Tables S1 and S2).

### 3.2. Association between Total Cholesterol Variability and Osteoporotic Fractures

**Figure 2:**
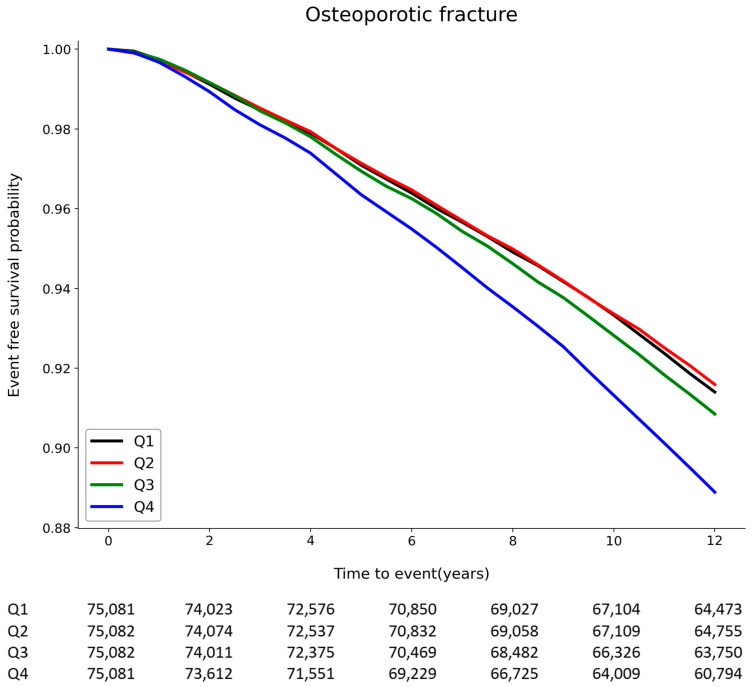
Kaplan–Meier survival curve displaying the estimated fracture-free probability for quartiles of total cholesterol variability.

There were 29,044 fracture events (9.67%) during a median follow-up of 11.6 ± 2.42 years. The incidence of osteoporotic fractures increased with higher TC variability, and there were significant stepwise relationships between osteoporotic fractures and increasing quartiles of TC variability (*p* < 0.001) (Figure 2). The Kaplan–Meier curves assessing different sites of osteoporotic fractures as endpoints showed similar results (Figures S1–S4). Considering multivariable Cox regression analysis, the highest and 3rd quartiles showed significantly increased risk compared with the lowest quartile based on the CV of TC variability, and a significantly positive trend of risk was noted with increasing quartiles (Table 2). The three indicators of TC variability showed similar results with the highest quartile displaying a 7–11% increased risk when compared to the lowest quartile according to the CV (HR 1.11, 95% CI [1.08–1.15]), SD (HR 1.07, 95% CI [1.04–1.11]), and VIM (HR 1.07, 95% CI [1.04–1.11]). The same results were observed in the landmark analysis (Table S3). Because the female sex is the risk factor for osteoporotic fractures and the sex ratio differs according to quartile in this study, a sex-stratified analysis was performed. Similar to the results of the analysis for all genders, both men and women in the highest quartile had a significantly higher risk of osteoporotic fractures compared to those in the lowest quartile according to the CV (HR for men 1.22, HR for women 1.06), SD (HR for men 1.16, HR for women 1.04), and VIM (HR for men 1.16, HR for women 1.05), showing larger associations in men than women (Tables S4 and S5). Furthermore, the significant tendency for the risk to increase as the quartile increased was observed in both men and women (*p*-value for trend <0.001). The osteoporotic fracture risk according to the deciles of TC variability was analyzed (Table S6), and a landmark analysis was also performed (Table S7). 

### 3.3. Subgroup Analyses

The subcategories of osteoporotic fractures were investigated for risk assessment. For vertebral fractures, which included the most events, there was a significant increase in HRs (HRs of 1.12 for CV, 1.07 for SD, 1.07 for VIM) when comparing the highest quartile to the lowest quartile, demonstrating a significant tendency (*p* < 0.001) to increased risk with increasing quartiles (Table S8). For hip fractures, the third common fracture site, all three measures of TC variability showed a significantly increased risk (HRs of 1.37 for CV, 1.22 for SD, 1.22 for VIM), greater than vertebral fractures, and a significantly increasing trend observed with increasing quartiles (Table S9). There were no significant associations between distal radius fractures or humerus fractures and TC variability based on the CV, SD, and VIM (Tables S10 and S11).

## 4. Discussion

We found a positive association between TC variability and osteoporotic fractures in this nationwide cohort study. During a long follow-up period of a mean of 11.6 years, high TC variability was significantly associated with the risk of osteoporotic fractures, and this significant association persisted after adjusting for potential confounders. Three different indices of TC variability showed consistent results. Among the various sites of fractures, a significantly increased fracture risk was observed in the spine and hip as the TC variability increased.

Several studies have been published elucidating the relationship between detailed lipid fraction levels and osteoporotic fractures, though the main culprit was different among these studies. One of them showed that serum lipid levels are associated with the existence of vertebral fracture rather than the BMD alterations, and the TC, TG, and LDL-C levels, especially TC levels, were significantly lower in postmenopausal women with a history of vertebral fractures than in the controls who did not have vertebral fractures [29]. In a prospective observational study of Swedish subjects, it has been demonstrated that high serum TC is an independent risk factor for osteoporotic fractures, and its predictive power increases over time [30]. On the other hand, in a cross-sectional study of Japanese postmenopausal women, it was suggested that increased serum LDL-C levels may play a role as a risk factor for non-vertebral fragility fractures independent of potential confounders, including bone-related biomarkers and vitamin D levels [31]. Another cross-sectional study conducted in China found a significant positive association between serum HDL-C levels and osteoporotic fractures in women, and a significant positive association between serum TG levels and osteoporotic fractures in both genders [32]. As mentioned above, the results of observational studies have been inconsistent about whether serum lipid levels are associated with fracture and which lipid fraction is the main factor. Given these limitations of studies showing conflicting results, absolute cholesterol levels at a particular time point may not be robust biomarkers due to the inherent variability of the values. This idea prompted us to evaluate TC variability as a risk factor for osteoporotic fractures.

Although the mechanism remains unclear, there has been evidence linking serum lipids and osteoporosis through a mechanism of atherosclerosis [33,34,35]. Several studies reported that trabecular bone density was independently associated with atherosclerotic burden, measured by arterial intima-media thickness or calcium scoring [36,37,38]. A previous study reporting a proportionally increased risk of cardiovascular outcome to the severity of osteoporosis in postmenopausal women, even after adjusting for potential confounders, suggests an association between osteoporosis and atherosclerotic burden [39]. Another explanation is that the differentiation of bone-forming cells can be inhibited due to lipid accumulation in the bone vessels, while osteoclastic differentiation can be promoted by oxidized lipids [20]. Meanwhile, statins may have a protective effect against osteoporosis, possibly by modulating receptor activator of nuclear factor kappa b ligand-osteoprotegerin (RANKL/OPG), which is a shared pathway among statin, osteoporosis, and adipogenesis [40]. In addition, statins have been shown to promote osteoblast differentiation and new bone formation by enhancing the expression of the bone morphogenetic protein-2 in vitro and rodents, and it has been suggested that these beneficial effects on bone can be obtained at doses similar to those used in humans [41].

The role of cholesterol variability in the occurrence of osteoporotic fractures is even more elusive. The mechanism of the effect of cholesterol variability on adverse health outcomes has been poorly understood, and the evidence has been suggested mainly from observational studies using big data. However, atherosclerosis may be suggested as a clue supporting the relationship between TC variability and osteoporotic fractures, as well as serum TC levels. A proposed mechanism for how cholesterol variability affects atherosclerosis is that variability in lipid efflux mechanisms and rebound endothelial dysfunction induced by high cholesterol variability may cause instability of the vessel wall [13,14,42], thereby increasing the atherosclerotic burden. Atheroma progression may adversely affect bone density and lead to an increase in subsequent osteoporotic fractures. However, it should also be noted that cholesterol variability could be an epiphenomenon of other conditions that contribute to elevated fracture risk and may not play a causal role [12].

### Limitations

The advantage of this study lies in the fact that it investigated the relationship between cholesterol variability and osteoporotic fractures in a population-based validated national database with a longitudinal setting. However, we acknowledge several limitations of our study. First, because measurements of LDL-C and HDL-C cholesterol levels were performed since 2008 and this study enrolled the data before 2008, the health examination dataset provides only total cholesterol levels and no information on lipid fractions. As is well known, TG, LDL-C, and HDL-C have different roles in the transport of cholesterol and different functions regarding atherogenesis and inflammation. Although the lack of detailed information on lipid fractions is one of the major limitations of this study, several previous studies have shown that total cholesterol variability is associated with the risk of vascular outcomes in patients after percutaneous coronary intervention, atrial fibrillation, end-stage renal disease, and dementia [15,18,43,44,45]. Second, the information about several medical conditions that can affect total cholesterol levels, including a history of familial hypercholesterolemia, and several well-known risk factors for osteoporotic fractures, including BMD values, vitamin-D levels, history of premature menopause, the use of medications such as glucocorticoids, and other endocrine or chronic inflammatory diseases, were not included in this study. Several protective factors including calcium intake and hormone replacement therapy were also not analyzed, as there are no previous studies on algorithms validating these data from the NHIS database. Information about breastfeeding, years since menopause, estrogen replacement therapy, and calcium supplements could be considered personal and sensitive, and although there have been previous studies that defined menopause indirectly as cases of hysterectomy or oophorectomy, or women aged 50 years or older [46,47], these methods have not been validated and are therefore not included in this study. Thus, the confounding effect caused by these factors cannot be completely excluded. The variability of other biological measures, such as glucose, was also not considered. Third, though the method has been used in previous studies, the diagnosis of comorbidities and osteoporotic fractures using ICD-10 codes may be inaccurate. Moreover, it is not possible to differentiate between fragility fractures due to low-energy trauma and osteoporotic fractures due to major trauma. Regarding comorbidities, laboratory results and a self-report questionnaire were also used to code the presence of comorbidities such as hypertension, diabetes mellitus, dyslipidemia, and renal disease (supplementary methods). Fourth, although atherosclerosis could be pointed to as a mediator linking TC variability with osteoporotic fractures, other test results that could evaluate the burden of atherosclerosis were not included in the national health examination. Therefore, the correlation between the severity of atherosclerosis and osteoporotic fractures could not be investigated. Fifth, because this is a retrospective study of a prospectively collected database, we cannot confirm the causal relationship between variables and outcomes or explain the underlying mechanism. It is necessary to prove a robust causal relationship rather than simply identify an epidemiological association for further research for clinical application.

## 5. Conclusions

Our study demonstrated that higher visit-to-visit variability in TC is associated with osteoporotic fractures, especially vertebral and hip fractures. Although the mechanism underlying this association is not well known, our finding suggests that intraindividual cholesterol variability may have a role as a biomarker for osteoporotic fracture risk. In addition, our study supports the possible role of cholesterol variability as a biomarker of metabolic diseases. Further studies are required to evaluate the role of specific cholesterol components with detailed cholesterol measurements including TG, LDL-C, and HDL-C, and to establish causality.

## Figures and Tables

**Table 1:** Baseline characteristics of subjects according to the quartiles of total cholesterol variability.

Variables	Total	Q1	Q2	Q3	Q4	*p*-Value
Number of participants (%)	300,326	75,081 (25.0)	75,082 (25.0)	75,082 (25.0)	75,081 (25.0)	
Age, years	56.25 ± 5.92	56.17 ± 5.89	55.75 ± 5.57	55.98 ± 5.69	57.11 ± 6.41	<0.001
Sex						<0.001
Male	221,339 (73.7)	57,057 (76.0)	57,313 (76.3)	55,397 (73.8)	51,572 (68.7)	
Female	78,987 (26.3)	18,024 (24.0)	17,769 (23.7)	19,685 (26.2)	23,509 (31.3)	
Body mass index (kg/m^2^)	23.98 ± 2.76	23.93 ± 2.73	23.91 ± 2.72	23.94 ± 2.75	24.13 ± 2.85	<0.001
Household income						<0.001
Q1, lowest	80,324 (26.7)	18,443 (24.6)	18,554 (24.7)	20,248 (27.0)	23,079 (30.7)	
Q2	83,315 (27.7)	19,805 (26.4)	20,194 (26.9)	21,123 (28.1)	22,193 (29.6)	
Q3	71,441 (23.8)	18,058 (24.1)	18,121 (24.1)	17,934 (23.9)	17,328 (23.1)	
Q4, highest	65,246 (21.7)	18,775 (25.0)	18,213 (24.3)	15,777 (21.0)	12,481 (16.6)	
Smoking status						<0.001
Never	179,488 (59.8)	43,722 (58.2)	43,499 (57.9)	45,013 (60.0)	47,254 (62.9)	
Former	47,434 (15.8)	12,676 (16.9)	12,429 (16.6)	11,625 (15.5)	10,704 (14.3)	
Current	73,404 (24.4)	18,683 (24.9)	19,154 (25.5)	18,444 (24.6)	17,123 (22.8)	
Alcohol consumption (days/week)						<0.001
None	200,543 (66.8)	49,662 (66.1)	49,269 (65.6)	49,959 (66.5)	51,653 (68.8)	
1–4	90,419 (30.1)	23,267 (31.0)	23,667 (31.5)	22,802 (30.4)	20,683 (27.6)	
≥5	9364 (3.1)	2152 (2.9)	2146 (2.9)	2321 (3.1)	2745 (3.7)	
Regular physical activity (days/week)						<0.001
None	121,585 (40.5)	29,165 (38.8)	29,600 (39.4)	30,347 (40.4)	32,473 (43.3)	
1–4	150,934 (50.3)	39,025 (52.0)	38,734 (51.6)	37,922 (50.5)	35,253 (47.0)	
≥5	27,807 (9.3)	6891 (9.2)	6748 (9.0)	6813 (9.1)	7355 (9.8)	
Comorbidities						
Hypertension	114,971 (38.3)	25,271 (33.7)	25,546 (34.0)	27,677 (36.9)	36,477 (48.6)	<0.001
Diabetes mellitus	58,940 (19.6)	11,809 (15.7)	12,276 (16.4)	13,823 (18.4)	21,032 (28.0)	<0.001
Dyslipidemia	93,928 (31.3)	16,905 (22.5)	18,251 (24.3)	22,175 (29.5)	36,597 (48.7)	<0.001
Stroke	7880 (2.6)	1479 (2.0)	1487 (2.0)	1778 (2.4)	3136 (4.2)	<0.001
Atrial fibrillation	2739 (0.9)	555 (0.7)	521 (0.7)	618 (0.8)	1045 (1.4)	<0.001
Renal disease	7700 (2.6)	1386 (1.9)	1445 (1.9)	1707 (2.3)	3162 (4.2)	<0.001
Cancer	13,489 (4.5)	2942 (3.9)	3072 (4.1)	3305 (4.4)	4170 (5.6)	<0.001
On lipid-lowering agents	29,287 (9.8)	5410 (7.2)	5658 (7.5)	6874 (9.2)	11,345 (15.1)	<0.001
Mean TC (mg/dL)	198.66 ± 31.16	199.22 ± 28.61	198.02 ± 28.52	197.56 ± 29.09	199.84 ± 37.44	<0.001
TC variability						
CV (%)	10.11 ± 6.13	4.8 ± 1.3	7.81 ± 0.72	10.56 ± 0.93	17.28 ± 7.89	<0.001
SD	20.41 ± 23.66	9.57 ± 2.94	15.54 ± 2.65	20.89 ± 3.59	35.71 ± 42.84	<0.001
VIM (%)	21.22 ± 22.53	9.46 ± 2.86	15.45 ± 2.54	20.85± 3.51	35.68 ± 42.79	<0.001

*p*-value by Chi-square test. Data are expressed as the mean ± SD, or n (%). Q, quartile; TC, total cholesterol; CV, coefficient of variation; SD, standard deviation; VIM, variability independent of the mean.

**Table 2:** The risk for the occurrence of osteoporotic fractures according to the quartiles of total cholesterol variability.

						Multivariable Model (1)			Multivariable Model (2)		
	Number of Participants	Number of Events	Event Rate (%) (95% CI)	Person-Years	Incidence Rate (Per 1000 Person-Years)	Adjusted HR (95% CI)	*p*-Value	*p*-Value for Trend	Adjusted HR (95% CI)	*p*-Value	*p*-Value for Trend
CV								<0.001			<0.001
Q1	75,081	6704	8.93 (8.72, 9.14)	874,299.98	7.67	1 (reference)			1 (reference)		
Q2	75,082	6603	8.79 (8.58, 9.01)	874,827.89	7.55	1.02 (0.99, 1.06)	0.230		1.02 (0.99, 1.05)	0.282	
Q3	75,082	7121	9.48 (9.26, 9.70)	869,543.07	8.19	1.06 (1.03, 1.10)	<0.001		1.06 (1.02, 1.09)	0.001	
Q4	75,081	8616	11.48 (11.23, 11.72)	852,157.80	10.11	1.11 (1.08, 1.15)	<0.001		1.10 (1.07, 1.14)	<0.001	
SD								<0.001			<0.001
Q1	75,095	6767	9.01 (8.80, 9.23)	872,764.30	7.75	1 (reference)			1 (reference)		
Q2	75,062	6522	8.69 (8.48, 8.90)	874,326.38	7.46	0.99 (0.95, 1.02)	0.459		0.99 (0.96, 1.03)	0.724	
Q3	75,087	7117	9.48 (9.26, 9.70)	869,819.28	8.18	1.03 (0.99, 1.06)	0.153		1.04 (1.00, 1.07)	0.027	
Q4	75,082	8638	11.50 (11.26, 11.75)	853,918.79	10.12	1.07 (1.04, 1.11)	<0.001		1.10 (1.06, 1.13)	<0.001	
VIM								<0.001			<0.001
Q1	75,081	6767	9.01 (8.80, 9.23)	872,602.28	7.75	1 (reference)			1 (reference)		
Q2	75,082	6522	8.69 (8.48, 8.90)	874,563.86	7.46	0.99 (0.95, 1.02)	0.444		0.99 (0.96, 1.03)	0.704	
Q3	75,082	7117	9.48 (9.26, 9.70)	869,749.16	8.18	1.03 (0.99, 1.06)	0.156		1.04 (1.00, 1.07)	0.027	
Q4	75,081	8638	11.50 (11.26, 11.75)	853,913.44	10.12	1.07 (1.04, 1.11)	<0.001		1.10 (1.06, 1.13)	<0.001	

Multivariable model (1) was adjusted for age, sex, body mass index, income levels, smoking, alcohol consumption, regular physical activity, hypertension, diabetes mellitus, dyslipidemia, stroke, atrial fibrillation, renal disease, cancer, and on a lipid-lowering agent. Multivariable model (2) was adjusted for age, sex, body mass index, income levels, smoking, alcohol consumption, regular physical activity, hypertension, diabetes mellitus, dyslipidemia, stroke, atrial fibrillation, renal disease, cancer, on a lipid-lowering agent, and mean TC. HR, hazard ratio; CI, confidence interval; CV, coefficient of variation; Q, quartile; SD, standard deviation; VIM, variability independent of the mean.

## Data Availability

This study used the data from the National Health Insurance Service-National Health Screening Cohort (NHIS-HEALS) database after permission through the following website [http://nhiss.nhis.or.kr/bd/ab/bdaba021eng.do] access date (12 August 2022).

## Supplementary Materials

The following supporting information can be downloaded at: https://www.mdpi.com/article/10.3390/jpm13030509/s1, Figure S1: Kaplan–Meier survival curve displaying the estimated vertebral fracture-free probability. Figure S2: Kaplan–Meier survival curve displaying the estimated hip fracture-free probability. Figure S3: Kaplan–Meier survival curve displaying the estimated distal radius fracture-free probability. Figure S4: Kaplan–Meier survival curve displaying the estimated humerus fracture-free probability; Table S1. Risk factors for the occurrence of osteoporotic fractures. Table S2. Risk factors for the occurrence of osteoporotic fractures (landmark analysis). Table S3. The risk for the occurrence of osteoporotic fractures according to the quartiles of total cholesterol variability (landmark analysis). Table S4. The risk for the occurrence of osteoporotic fractures according to the quartiles of total cholesterol variability in men. Table S5. The risk for the occurrence of osteoporotic fractures according to the quartiles of total cholesterol variability in women. Table S6. The risk for the occurrence of osteoporotic fractures according to the deciles of total cholesterol variability. Table S7. The risk for the occurrence of osteoporotic fracture according to the deciles of total cholesterol variability (landmark analysis). Table S8. The risk for the occurrence of vertebral fractures according to the quartiles of total cholesterol variability. Table S9. The risk for the occurrence of hip fractures according to the quartiles of total cholesterol variability. Table S10. The risk for the occurrence of distal radius fractures according to the quartiles of total cholesterol variability. Table S11. The risk for the occurrence of humerus fractures according to the quartiles of total cholesterol variability.
