# Supplementary material for: Total Cholesterol Variability and the Risk of Osteoporotic Fractures: A Nationwide Population-Based Cohort Study
Source: J Pers Med. 2023 Mar 11;13(3):509. doi: 10.3390/jpm13030509 (PMC10054569; doi:10.3390/jpm13030509)
Supplement: Supplementary file 1 [file jpm-13-00509-s001.zip › 2022-11-14 TC-osteoporotic_Fx_Supplementary methods.pdf]

Supplementary Methods

Hypertension was defined as satisfying one of the following criteria:

1) at least one claim of diagnostic codes (International Classification of Diseases, Tenth Revision [ICD-10] I10–15) with the prescription of an antihypertensive agent, 2) two or more claims of diagnostic codes (ICD-10 codes E11–14), 3) systolic/diastolic blood pressure ≥ 140/90 mmHg, or 4) self-reported hypertension in the questionnaire.

Diabetes mellitus was defined as satisfying one of the following criteria:

1) at least one claim of diagnostic codes (ICD-10 codes E11–14) with the prescription of an antidiabetic agent, 2) two or more claims of diagnostic codes (ICD-10 codes E11–14), 3) fasting serum glucose level ≥ 7.0 mmol/L, or 4) self-reported diabetes mellitus in the questionnaire.

Dyslipidemia was defined as satisfying one of the following criteria: 1) at least one claim of diagnostic codes (ICD-10 code E78) with the prescription of a dyslipidemia-related agent, including statins, 2) two or more claims of diagnostic codes (ICD-10 code E78), 3) TC ≥ 240 mg/dL.

Stroke was defined as two or more claims of diagnostic code (ICD-10 codes I60–63) with brain CT/MRI and hospital admission.

Atrial fibrillation was defined as two or more claims of diagnostic code (ICD-10 code I48).

Renal disease was defined as two or more claims of diagnostic codes (ICD-10 codes N17-19, I12-13, E082, E102, E112, E132), or estimated glomerular filtration rate less than 60 mL/min/1.73 m^2.

Cancer was defined as having one admission or at least three outpatient claims of diagnostic code (ICD-10 codes C00–97) with specific registration code of ‘V027’ or ‘V193–4’
