# Supplementary material for: Total Cholesterol Variability and the Risk of Osteoporotic Fractures: A Nationwide Population-Based Cohort Study
Source: J Pers Med. 2023 Mar 11;13(3):509. doi: 10.3390/jpm13030509 (PMC10054569; doi:10.3390/jpm13030509)
Supplement: Supplementary file 1 [file jpm-13-00509-s001.zip › 2023-02-27 TC-osteoporotic_Fx_supplementary tables.pdf]

Table S1. Risk factors for the occurrence of osteoporotic fractures.

			Multivariable model (1)		Multivariable model (2)	
Variables	Crude HR (95% CI)	*P*-value	Adjusted HR (95% CI)	*P*-value	Adjusted HR (95% CI)	*P*-value
Age, years	1.08 (1.07, 1.08)	<.001	1.07 (1.06, 1.07)	<.001	1.07 (1.06, 1.07)	<.001
Sex						
Male	1 (reference)		1 (reference)		1 (reference)	
Female	2.93 (2.86, 2.99)	<.001	2.74 (2.66, 2.83)	<.001	2.78 (2.69, 2.86)	<.001
Body mass index (kg/m^2)	0.97 (0.97, 0.98)	<.001	0.98 (0.97, 0.98)	<.001	0.98 (0.98, 0.98)	<.001
Household income						
Q1, lowest	1 (reference)		1 (reference)		1 (reference)	
Q2	0.81 (0.79, 0.83)	<.001	1.00 (0.97, 1.03)	0.964	1.00 (0.97, 1.03)	0.965
Q3	0.66 (0.64, 0.68)	<.001	0.96 (0.93, 0.99)	0.010	0.96 (0.93, 0.99)	0.008
Q4, highest	0.45 (0.44, 0.47)	<.001	0.80 (0.77, 0.83)	<.001	0.80 (0.77, 0.83)	<.001
Smoking status						
Never	1 (reference)		1 (reference)		1 (reference)	
Former	0.48 (0.47, 0.50)	<.001	0.96 (0.91, 1.00)	0.041	0.96 (0.92, 1.00)	0.053
Current	0.59 (0.57, 0.61)	<.001	1.12 (1.08, 1.17)	<.001	1.13 (1.09, 1.17)	<.001
Alcohol consumption (days/week)						
None	1 (reference)		1 (reference)		1 (reference)	
1-4	0.62 (0.60, 0.64)	<.001	1.04 (1.01, 1.08)	0.007	1.05 (1.01, 1.08)	0.006
≥ 5	1.00 (0.94, 1.07)	0.934	1.35 (1.26, 1.44)	<.001	1.35 (1.26, 1.44)	<.001
Regular physical activity (days/week)						
None	1 (reference)		1 (reference)		1 (reference)	
1-4	0.65 (0.63, 0.66)	<.001	0.92 (0.90, 0.94)	<.001	0.92 (0.90, 0.94)	<.001
≥ 5	0.83 (0.80, 0.87)	<.001	0.93 (0.90, 0.97)	0.001	0.93 (0.90, 0.97)	0.001
Comorbidities						
Hypertension	1.25 (1.23, 1.28)	<.001	1.01 (0.98, 1.03)	0.667	1.00 (0.98, 1.03)	0.898
Diabetes mellitus	1.22 (1.18, 1.25)	<.001	1.07 (1.04, 1.11)	<.001	1.07 (1.04, 1.10)	<.001
Dyslipidemia	1.21 (1.19, 1.24)	<.001	1.00 (0.97, 1.03)	0.910	1.04 (1.01, 1.07)	0.009
Stroke	1.73 (1.64, 1.84)	<.001	1.22 (1.15, 1.29)	<.001	1.21 (1.14, 1.28)	<.001
Atrial fibrillation	1.38 (1.24, 1.54)	<.001	1.17 (1.05, 1.30)	0.004	1.15 (1.04, 1.28)	0.009
Renal disease	1.29 (1.21, 1.38)	<.001	1.07 (1.00, 1.14)	0.061	1.05 (0.98, 1.13)	0.134
Cancer	1.29 (1.22, 1.35)	<.001	1.17 (1.11, 1.23)	<.001	1.16 (1.10, 1.22)	<.001
On lipid-lowering agent	0.89 (0.82, 0.96)	<.001	0.92 (0.87, 0.97)	0.002	0.91 (0.88, 0.94)	0.007
Mean TC (mg/dL)	1.03 (1.01, 1.05)	0.005			1.02 (1.01, 1.04)	<.001
TC variability						
CV (%)						
Q1	1 (reference)		1 (reference)		1 (reference)	
Q2	1.02 (0.99, 1.05)	0.238	1.02 (0.99, 1.06)	0.230	1.02 (0.99, 1.05)	0.282
Q3	1.06 (1.02, 1.09)	<.001	1.06 (1.03, 1.10)	<.001	1.06 (1.02, 1.09)	0.001
Q4	1.12 (1.08, 1.16)	<.001	1.11 (1.08, 1.15)	<.001	1.10 (1.07, 1.14)	<.001

SD						
Q1	1 (reference)		1 (reference)		1 (reference)	
Q2	0.99 (0.94, 1.03)	0.582	0.99 (0.95, 1.02)	0.459	0.99 (0.96, 1.03)	0.724
Q3	1.04 (1.00, 1.06)	0.067	1.03 (0.99, 1.06)	0.153	1.04 (1.00, 1.07)	0.027
Q4	1.12 (1.04, 1.20)	<.001	1.07 (1.04, 1.11)	<.001	1.10 (1.06, 1.13)	<.001
VIM (%)						
Q1	1 (reference)		1 (reference)		1 (reference)	
Q2	0.99 (0.94, 1.03)	0.682	0.99 (0.95, 1.02)	0.444	0.99 (0.96, 1.03)	0.704
Q3	1.03 (1.00, 1.05)	0.053	1.03 (0.99, 1.06)	0.156	1.04 (1.00, 1.07)	0.027
Q4	1.12 (1.03, 1.21)	<.001	1.07 (1.04, 1.11)	<.001	1.10 (1.06, 1.13)	<.001

Multivariable model (1) was adjusted for age, sex, body mass index, income levels, smoking, alcohol consumption, regular physical activity, hypertension, diabetes mellitus, dyslipidemia, stroke, atrial fibrillation, renal disease, cancer, and on lipid-lowering agent.
Multivariable model (2) was adjusted for age, sex, body mass index, income levels, smoking, alcohol consumption, regular physical activity, hypertension, diabetes mellitus, dyslipidemia, stroke, atrial fibrillation, renal disease, cancer, on lipid-lowering agent, and mean TC.
HR, hazard ratio; CI, confidence interval; Q, quartile; TC, total cholesterol; CV, coefficient of variation; SD, standard deviation; VIM, variability independent of the mean.

Table S2. Risk factors for the occurrence of osteoporotic fractures (landmark analysis).

			Multivariable model (1)		Multivariable model (2)	
Variables	Crude HR (95% CI)	*P*-value	Adjusted HR (95% CI)	*P*-value	Adjusted HR (95% CI)	*P*-value
Age, years	1.08 (1.07, 1.08)	<.001	1.07 (1.06, 1.07)	<.001	1.07 (1.06, 1.07)	<.001
Sex						
Male	1 (reference)		1 (reference)		1 (reference)	
Female	2.94 (2.87, 3.01)	<.001	2.75 (2.67, 2.84)	<.001	2.79 (2.70, 2.87)	<.001
Body mass index (kg/m2)	0.97 (0.97, 0.98)	<.001	0.98 (0.97, 0.98)	<.001	0.98 (0.98, 0.98)	<.001
Household income						
Q1, lowest	1 (reference)		1 (reference)		1 (reference)	
Q2	0.81 (0.78, 0.83)	<.001	1.00 (0.97, 1.03)	0.948	1.00 (0.97, 1.03)	0.977
Q3	0.66 (0.64, 0.68)	<.001	0.96 (0.93, 0.99)	0.009	0.96 (0.93, 0.99)	0.007
Q4, highest	0.45 (0.44, 0.47)	<.001	0.80 (0.77, 0.83)	<.001	0.80 (0.77, 0.83)	<.001
Smoking status						
Never	1 (reference)		1 (reference)		1 (reference)	
Former	0.48 (0.47, 0.50)	<.001	0.96 (0.92, 1.00)	0.058	0.96 (0.92, 1.00)	0.075
Current	0.59 (0.57, 0.61)	<.001	1.12 (1.08, 1.17)	<.001	1.13 (1.09, 1.17)	<.001
Alcohol consumption (days/week)						
None	1 (reference)		1 (reference)		1 (reference)	
1-4	0.62 (0.60, 0.64)	<.001	1.05 (1.01, 1.08)	0.006	1.05 (1.01, 1.08)	0.005
≥ 5	0.99 (0.93, 1.06)	0.807	1.34 (1.25, 1.43)	<.001	1.33 (1.25, 1.42)	<.001
Regular physical activity (days/week)						
None	1 (reference)		1 (reference)		1 (reference)	
1-4	0.64 (0.63, 0.66)	<.001	0.92 (0.89, 0.94)	<.001	0.92 (0.89, 0.94)	<.001
≥ 5	0.83 (0.80, 0.87)	<.001	0.93 (0.90, 0.97)	0.001	0.93 (0.90, 0.97)	0.001
Comorbidities						
Hypertension	1.26 (1.23, 1.29)	<.001	1.01 (0.98, 1.03)	0.631	1.00 (0.98, 1.03)	0.867
Diabetes mellitus	1.22 (1.19, 1.25)	<.001	1.07 (1.04, 1.11)	<.001	1.07 (1.03, 1.10)	<.001
Dyslipidemia	1.22 (1.19, 1.25)	<.001	1.00 (0.97, 1.03)	0.992	1.04 (1.01, 1.07)	0.005
Stroke	1.73 (1.63, 1.83)	<.001	1.21 (1.14, 1.29)	<.001	1.20 (1.13, 1.28)	<.001
Atrial fibrillation	1.38 (1.24, 1.53)	<.001	1.16 (1.05, 1.30)	0.006	1.15 (1.03, 1.28)	0.012
Renal disease	1.29 (1.22, 1.36)	<.001	1.17 (1.11, 1.23)	<.001	1.16 (1.10, 1.22)	<.001
Cancer	1.30 (1.22, 1.39)	<.001	1.07 (1.00, 1.15)	0.047	1.06 (0.99, 1.13)	0.110
On lipid-lowering agent	0.87 (0.80, 0.94)	<.001	0.91 (0.85, 0.97)	0.002	0.92 (0.88, 0.96)	0.003
Mean TC (mg/dL)	1.03 (1.01, 1.05)	0.005			1.02 (1.01, 1.04)	<.001
TC variability						
CV (%)	0.98 (0.95, 1.01)	0.244	1.02 (0.98, 1.05)	0.352	1.01 (0.98, 1.05)	0.423
SD	1.07 (1.03, 1.10)	<.001	1.06 (1.03, 1.10)	0.001	1.06 (1.02, 1.09)	0.002
VIM (%)	1.32 (1.28, 1.36)	<.001	1.11 (1.08, 1.15)	<.001	1.10 (1.07, 1.14)	<.001

Multivariable model (1) was adjusted for age, sex, body mass index, income levels, smoking, alcohol consumption, regular physical activity, hypertension, diabetes mellitus, dyslipidemia, stroke, atrial fibrillation, renal disease, cancer, and on lipid-lowering agent.

Multivariable model (2) was adjusted for age, sex, body mass index, income levels, smoking, alcohol consumption, regular physical activity, hypertension, diabetes mellitus, dyslipidemia, stroke, atrial fibrillation, renal disease, cancer, on lipid-lowering agent, and mean TC.
HR, hazard ratio; CI, confidence interval; TC, total cholesterol; Q, quartile; CV, coefficient of variation; SD, standard deviation; VIM, variability independent of the mean.

Table S3. The risk for the occurrence of osteoporotic fractures according to the quartiles of total cholesterol variability (landmark analysis).

	Number of participants	Number of events	Event rate (%) (95% CI)	Person-years	Incidence rate (per 1000 person-years)	Multivariable model (1) Adjusted HR (95% CI)	*P*-value	*P*-value for trend	Multivariable model (2) Adjusted HR (95% CI)	*P*-value	*P*-value for trend
CV								<.001			<.001
Q1	75081	6591	8.78 (8.57, 8.99)	875683.04	7.53	1 (reference)			1 (reference)		
Q2	75082	6465	8.61 (8.40, 8.82)	876409.62	7.38	1.02 (0.98, 1.05)	0.352		1.01 (0.98, 1.05)	0.423	
Q3	75082	6993	9.31 (9.10, 9.53)	871033.92	8.03	1.06 (1.03, 1.10)	0.001		1.06 (1.02, 1.09)	0.002	
Q4	75081	8472	11.28 (11.04, 11.52)	853877.07	9.92	1.11 (1.08, 1.15)	<.001		1.10 (1.07, 1.14)	<.001	
SD								<.001			<.001
Q1	75095	6655	8.86 (8.65, 9.08)	874077.07	7.61	1 (reference)			1 (reference)		
Q2	75062	6383	8.50 (8.30, 8.71)	875947.61	7.29	0.98 (0.95, 1.02)	0.297		0.99 (0.96, 1.02)	0.520	
Q3	75087	6993	9.31 (9.09, 9.53)	871289.96	8.03	1.02 (0.99, 1.06)	0.180		1.04 (1.00, 1.07)	0.031	
Q4	75082	8490	11.31 (11.07, 11.55)	855689.00	9.92	1.07 (1.03, 1.11)	<.001		1.10 (1.06, 1.13)	<.001	
VIM								<.001			<.001
Q1	75081	6655	8.86 (8.65, 9.08)	873915.06	7.62	1 (reference)			1 (reference)		
Q2	75082	6383	8.50 (8.29, 8.71)	876185.09	7.28	0.98 (0.95, 1.02)	0.285		0.99 (0.96, 1.02)	0.503	
Q3	75082	6993	9.31 (9.10, 9.53)	871219.84	8.03	1.02 (0.99, 1.06)	0.183		1.04 (1.00, 1.07)	0.032	
Q4	75081	8490	11.31 (11.07, 11.55)	855683.66	9.92	1.07 (1.03, 1.11)	<.001		1.10 (1.06, 1.13)	<.001	

Multivariable model (1) was adjusted for age, sex, body mass index, income levels, smoking, alcohol consumption, regular physical activity, hypertension, diabetes mellitus, dyslipidemia, stroke, atrial fibrillation, renal disease, cancer, and on lipid-lowering agent.

Multivariable model (2) was adjusted for age, sex, body mass index, income levels, smoking, alcohol consumption, regular physical activity, hypertension, diabetes mellitus, dyslipidemia, stroke, atrial fibrillation, renal disease, cancer, on lipid-lowering agent, and mean TC.

HR, hazard ratio; CI, confidence interval; CV, coefficient of variation; Q, quartile; SD, standard deviation; VIM, variability independent of the mean; TC, total cholesterol variability.

Table S4. The risk for the occurrence of osteoporotic fractures according to the quartiles of total cholesterol variability in men.

						Multivariable model (1)			Multivariable model (2)		
	Number of participants	Number of events	Event rate (%) (95% CI)	Person-years	Incidence rate (per 1000 person-years)	Adjusted HR (95% CI)	*P*-value	*P*-value for trend	Adjusted HR (95% CI)	*P*-value	*P*-value for trend
CV								<.001			<.001
Q1	57057	1756	3.08 (2.94, 3.22)	679989.84	2.58	1 (reference)			1 (reference)		
Q2	57313	1808	3.15 (3.01, 3.30)	682167.60	2.65	1.05 (0.99, 1.12)	0.130		1.05 (0.99, 1.13)	0.126	
Q3	55397	1895	3.42 (3.27, 3.57)	655800.65	2.89	1.10 (1.03, 1.18)	0.004		1.10 (1.03, 1.17)	0.005	
Q4	51572	2194	4.25 (4.08, 4.43)	597762.71	3.67	1.22 (1.15, 1.31)	<.001		1.22 (1.14, 1.30)	<.001	
SD								<.001			<.001
Q1	57927	1890	3.26 (3.12, 3.41)	688682.71	2.74	1 (reference)			1 (reference)		
Q2	57691	1804	3.13 (2.98, 3.27)	685815.45	2.63	0.99 (0.93, 1.06)	0.822		1.01 (0.94, 1.07)	0.857	
Q3	55227	1875	3.40 (3.24, 3.55)	653656.47	2.87	1.04 (0.98, 1.11)	0.238		1.06 (1.00, 1.14)	0.064	
Q4	50494	2084	4.13 (3.95, 4.30)	587566.19	3.54	1.16 (1.08, 1.23)	<.001		1.21 (1.13, 1.29)	<.001	
VIM								<.001			<.001
Q1	57916	1890	3.26 (3.12, 3.41)	688558.18	2.74	1 (reference)			1 (reference)		
Q2	57708	1804	3.13 (2.98, 3.27)	686015.02	2.63	0.99 (0.93, 1.06)	0.813		1.01 (0.94, 1.07)	0.867	
Q3	55221	1875	3.40 (3.24, 3.55)	653581.42	2.87	1.04 (0.98, 1.11)	0.240		1.06 (1.00, 1.14)	0.065	
Q4	50494	2084	4.13 (3.95, 4.30)	587566.19	3.55	1.16 (1.08, 1.23)	<.001		1.21 (1.13, 1.29)	<.001	

Multivariable model (1) was adjusted for age, sex, body mass index, income levels, smoking, alcohol consumption, regular physical activity, hypertension, diabetes mellitus, dyslipidemia, stroke, atrial fibrillation, renal disease, cancer, and on lipid-lowering agent.

Multivariable model (2) was adjusted for age, sex, body mass index, income levels, smoking, alcohol consumption, regular physical activity, hypertension, diabetes mellitus, dyslipidemia, stroke, atrial fibrillation, renal disease, cancer, on lipid-lowering agent, and mean TC.

HR, hazard ratio; CI, confidence interval; CV, coefficient of variation; Q, quartile; SD, standard deviation; VIM, variability independent of the mean.

Table S5. The risk for the occurrence of osteoporotic fractures according to the quartiles of total cholesterol variability in women

						Multivariable model (1)			Multivariable model (2)		
	Number of participants	Number of events	Event rate (%) (95% CI)	Person-years	Incidence rate (per 1000 person-years)	Adjusted HR (95% CI)	*P*-value	*P*-value for trend	Adjusted HR (95% CI)	*P*-value	*P*-value for trend
CV								<.001			<.001
Q1	18024	1865	10.35 (9.90, 10.79)	208827.66	8.93	1 (reference)			1 (reference)		
Q2	17769	1690	9.51 (9.08, 9.94)	207361.53	8.15	0.98 (0.92, 1.05)	0.524		0.97 (0.91, 1.04)	0.348	
Q3	19685	1910	9.70 (9.29, 10.12)	229522.68	8.32	1.02 (0.96, 1.09)	0.553		1.01 (0.95, 1.08)	0.805	
Q4	23509	2606	11.09 (10.68, 11.49)	271365.73	9.60	1.06 (1.02, 1.10)	0.003		1.05 (1.02, 1.08)	0.002	
SD								<.001			<.001
Q1	17168	1800	10.48 (10.03, 10.94)	198575.39	9.06	1 (reference)			1 (reference)		
Q2	17371	1637	9.42 (8.99, 9.86)	202998.12	8.06	0.94 (0.88, 1.01)	0.074		0.94 (0.87, 1.00)	0.054	
Q3	19860	1864	9.38 (8.98, 9.79)	231923.82	8.04	0.95 (0.89, 1.01)	0.088		0.95 (0.89,1.02)	0.127	
Q4	24588	2770	11.27 (10.87, 11.66)	283580.26	9.77	1.04 (1.01, 1.07)	0.046		1.04 (1.02, 1.06)	0.045	
VIM								<.001			<.001
Q1	17165	1800	10.49 (10.03, 10.94)	198537.91	9.07	1 (reference)			1 (reference)		
Q2	17374	1637	9.42 (8.99, 9.86)	203036.03	8.06	0.94 (0.88, 1.01)	0.072		0.94 (0.88, 1.01)	0.052	
Q3	19861	1864	9.39 (8.98, 9.79)	231928.75	8.04	0.95 (0.89, 1.01)	0.087		0.95 (0.89, 1.01)	0.125	
Q4	24587	2770	11.27 (10.87, 11.66)	283574.92	9.77	1.05 (1.02, 1.08)	0.045		1.05 (1.02, 1.07)	0.044	

Multivariable model (1) was adjusted for age, sex, body mass index, income levels, smoking, alcohol consumption, regular physical activity, hypertension, diabetes mellitus, dyslipidemia, stroke, atrial fibrillation, renal disease, cancer, and on lipid-lowering agent.

Multivariable model (2) was adjusted for age, sex, body mass index, income levels, smoking, alcohol consumption, regular physical activity, hypertension, diabetes mellitus, dyslipidemia, stroke, atrial fibrillation, renal disease, cancer, on lipid-lowering agent, and mean TC.

HR, hazard ratio; CI, confidence interval; CV, coefficient of variation; Q, quartile; SD, standard deviation; VIM, variability independent of the mean.

Table S6. The risk for the occurrence of osteoporotic fractures according to the deciles of total cholesterol variability.

						Multivariable model (1)			Multivariable model (2)		
	Number of participants	Number of events	Event rate (%) (95% CI)	Person-years	Incidence rate (per 1000 person-years)	Adjusted HR (95% CI)	*P*-value	*P*-value for trend	Adjusted HR (95% CI)	*P*-value	*P*-value for trend
CV								<.001			<.001
D1	30033	2876	9.58 (9.23, 9.93)	348328.08	8.26	1 (reference)			1 (reference)		
D2	30032	2563	8.53 (8.20, 8.86)	350370.05	7.32	1.00 (0.95, 1.06)	0.922		1.00 (0.95, 1.06)	0.917	
D3	30033	2630	8.76 (8.42, 9.09)	350459.76	7.50	1.04 (0.99, 1.10)	0.124		1.04 (0.99, 1.10)	0.131	
D4	30032	2611	8.69 (8.36, 9.03)	350449.47	7.45	1.02 (0.96, 1.07)	0.579		1.01 (0.96, 1.07)	0.638	
D5	30033	2627	8.75 (8.41, 9.08)	349520.51	7.52	1.01 (0.96, 1.07)	0.703		1.01 (0.96, 1.06)	0.789	
D6	30033	2753	9.17 (8.82, 9.51)	348636.72	7.90	1.05 (1.00, 1.11)	0.062		1.05 (0.99, 1.10)	0.085	
D7	30032	2867	9.55 (9.20, 9.90)	347644.65	8.25	1.07 (1.01, 1.12)	0.015		1.06 (1.01, 1.12)	0.026	
D8	30033	3074	10.24 (9.87, 10.60)	345839.70	8.89	1.10 (1.05, 1.16)	<.001		1.10 (1.04, 1.15)	0.001	
D9	30033	3380	11.25 (10.87, 11.63)	342391.51	9.87	1.13 (1.07, 1.19)	<.001		1.12 (1.06, 1.18)	<.001	
D10	30032	3663	12.20 (11.80, 12.59)	337188.29	10.86	1.11 (1.05, 1.16)	<.001		1.10 (1.04, 1.15)	<.001	
SD								<.001			<.001
D1	29996	2839	9.46 (9.12, 9.81)	347561.49	8.17	1 (reference)			1 (reference)		
D2	30058	2612	8.69 (8.36, 9.02)	350220.34	7.46	1.03 (0.98, 1.09)	0.257		1.03 (0.98, 1.09)	0.221	
D3	30038	2625	8.74 (8.40, 9.07)	349617.67	7.51	1.04 (0.98, 1.09)	0.205		1.04 (0.99, 1.10)	0.143	
D4	30051	2567	8.54 (8.21, 8.87)	350576.72	7.32	1.00 (0.95, 1.06)	0.974		1.01 (0.96, 1.06)	0.759	
D5	30014	2646	8.82 (8.48, 9.15)	349114.46	7.58	1.00 (0.95, 1.06)	0.897		1.01 (0.96, 1.07)	0.604	
D6	30041	2773	9.23 (8.89, 9.57)	348828.96	7.95	1.05 (0.99, 1.10)	0.094		1.06 (1.01, 1.12)	0.030	
D7	30028	2844	9.47 (9.12, 9.82)	347476.51	8.18	1.04 (0.99, 1.09)	0.167		1.06 (1.00, 1.11)	0.047	

D8	30039	3061	10.19 (9.83, 10.55)	346104.49	8.84	1.07 (1.02, 1.13)	0.009		1.09 (1.04, 1.15)	0.001	
D9	30024	3466	11.54 (11.16, 11.93)	342274.55	10.13	1.13 (1.08, 1.19)	<.001		1.16 (1.10, 1.22)	<.001	
D10	30037	3611	12.02 (11.63, 12.41)	339053.56	10.65	1.06 (1.01, 1.11)	0.029		1.09 (1.04, 1.15)	0.001	
VIM								<.001			<.001
D1	30032	2846	9.48 (9.13, 9.82)	347937.97	8.18	1 (reference)			1 (reference)		
D2	30033	2608	8.68 (8.35, 9.02)	349951.01	7.45	1.03 (0.98, 1.09)	0.269		1.03 (0.98, 1.09)	0.235	
D3	30033	2624	8.74 (8.40, 9.07)	349578.79	7.51	1.03 (0.98, 1.09)	0.224		1.04 (0.99, 1.10)	0.158	
D4	30032	2563	8.53 (8.20, 8.86)	350373.26	7.32	1.00 (0.95, 1.05)	0.978		1.01 (0.95, 1.06)	0.807	
D5	30033	2648	8.82 (8.48, 9.15)	349325.10	7.58	1.00 (0.95, 1.06)	0.918		1.01 (0.96, 1.07)	0.625	
D6	30033	2775	9.24 (8.90, 9.58)	348720.27	7.96	1.05 (0.99, 1.10)	0.094		1.06 (1.01, 1.12)	0.030	
D7	30032	2842	9.46 (9.12, 9.81)	347535.01	8.18	1.04 (0.98, 1.09)	0.186		1.05 (1.00, 1.11)	0.054	
D8	30033	3061	10.19 (9.83, 10.55)	346029.05	8.85	1.07 (1.02, 1.13)	0.010		1.09 (1.04, 1.15)	0.001	
D9	30033	3467	11.54 (11.16, 11.93)	342376.50	10.13	1.13 (1.08, 1.19)	<.001		1.15 (1.10, 1.21)	<.001	
D10	30032	3610	12.02 (11.63, 12.41)	339001.77	10.65	1.06 (1.01, 1.11)	0.032		1.09 (1.04, 1.15)	0.001	

Multivariable model (1) was adjusted for age, sex, body mass index, income levels, smoking, alcohol consumption, regular physical activity, hypertension, diabetes mellitus, dyslipidemia, stroke, atrial fibrillation, renal disease, cancer, and on lipid-lowering agent.
Multivariable model (2) was adjusted for age, sex, body mass index, income levels, smoking, alcohol consumption, regular physical activity, hypertension, diabetes mellitus, dyslipidemia, stroke, atrial fibrillation, renal disease, cancer, on lipid-lowering agent, and mean TC.
HR, hazard ratio; CI, confidence interval; CV, coefficient of variation; D, decile; SD, standard deviation; VIM, variability independent of the mean.

Table S7. The risk for the occurrence of osteoporotic fracture according to the deciles of total cholesterol variability (landmark analysis).

	Number of participants	Number of events	Event rate (%) (95% CI)	Person-years	Incidence rate (per 1000 person-years)	Multivariable model (1) Adjusted HR (95% CI)	*P*-value	*P*-value for trend	Multivariable model (2) Adjusted HR (95% CI)	*P*-value	*P*-value for trend
CV								<.001			<.001
D1	30033	2828	9.42 (9.07, 9.76)	348898.27	8.11	1 (reference)			1 (reference)		
D2	30032	2523	8.40 (8.07, 8.73)	350883.32	7.19	1.00 (0.95, 1.06)	0.878		1.00 (0.95, 1.06)	0.875	
D3	30033	2577	8.58 (8.25, 8.91)	351072.02	7.34	1.04 (0.99, 1.10)	0.163		1.04 (0.98, 1.10)	0.172	
D4	30032	2561	8.53 (8.20, 8.86)	350967.97	7.30	1.01 (0.96, 1.07)	0.624		1.01 (0.96, 1.07)	0.688	
D5	30033	2567	8.55 (8.22, 8.88)	350271.07	7.33	1.00 (0.95, 1.06)	0.923		1.00 (0.95, 1.05)	0.982	
D6	30033	2700	8.99 (8.65, 9.33)	349265.61	7.73	1.05 (0.99, 1.11)	0.080		1.04 (0.99, 1.10)	0.109	
D7	30032	2818	9.38 (9.04, 9.73)	348209.89	8.09	1.07 (1.01, 1.12)	0.017		1.06 (1.01, 1.12)	0.028	
D8	30033	3018	10.05 (9.69, 10.41)	346453.37	8.71	1.10 (1.05, 1.16)	<.001		1.09 (1.04, 1.15)	0.001	
D9	30033	3324	11.07 (10.69, 11.44)	343130.58	9.69	1.13 (1.07, 1.19)	<.001		1.12 (1.06, 1.18)	<.001	
D10	30032	3605	12.00 (11.61, 12.40)	337851.54	10.67	1.11 (1.05, 1.16)	<.001		1.10 (1.04, 1.15)	<.001	
SD								<.001			<.001
D1	29996	2794	9.31 (8.97, 9.66)	348119.40	8.03	1 (reference)			1 (reference)		
D2	30058	2570	8.55 (8.22, 8.88)	350698.78	7.33	1.03 (0.98, 1.09)	0.258		1.03 (0.98, 1.09)	0.221	
D3	30038	2582	8.60 (8.26, 8.93)	350135.60	7.37	1.04 (0.98, 1.09)	0.213		1.04 (0.99, 1.10)	0.146	
D4	30051	2503	8.33 (8.00, 8.66)	351316.40	7.12	0.99 (0.94, 1.05)	0.742		1.00 (0.95, 1.05)	0.965	
D5	30014	2589	8.63 (8.29, 8.96)	349754.51	7.40	1.00 (0.95, 1.05)	0.924		1.01 (0.96, 1.06)	0.757	
D6	30041	2726	9.07 (8.73, 9.41)	349374.49	7.80	1.05 (0.99, 1.10)	0.103		1.06 (1.01, 1.12)	0.032	
D7	30028	2796	9.31 (8.97, 9.66)	348076.90	8.03	1.04 (0.98, 1.09)	0.189		1.05 (1.00, 1.11)	0.052	

D8	30039	3000	9.99 (9.63, 10.34)	346803.64	8.65	1.07 (1.01, 1.12)	0.017		1.09 (1.03, 1.14)	0.002	
D9	30024	3406	11.34 (10.96, 11.73)	342988.89	9.93	1.13 (1.07, 1.19)	<.001		1.15 (1.10, 1.21)	<.001	
D10	30037	3555	11.84 (11.45, 12.22)	339735.05	10.46	1.06 (1.00, 1.11)	0.034		1.09 (1.04, 1.15)	0.001	
VIM								<.001			<.001
D1	30032	2801	9.33 (8.98, 9.67)	348495.87	8.04	1 (reference)			1 (reference)		
D2	30033	2566	8.54 (8.21, 8.87)	350429.45	7.32	1.03 (0.98, 1.09)	0.272		1.03 (0.98, 1.09)	0.236	
D3	30033	2581	8.59 (8.26, 8.93)	350096.73	7.37	1.03 (0.98, 1.09)	0.233		1.04 (0.99, 1.10)	0.162	
D4	30032	2499	8.32 (7.99, 8.65)	351112.94	7.12	0.99 (0.94, 1.04)	0.695		1.00 (0.95, 1.05)	0.913	
D5	30033	2591	8.63 (8.29, 8.96)	349965.16	7.40	1.00 (0.95, 1.05)	0.903		1.01 (0.96, 1.06)	0.781	
D6	30033	2728	9.08 (8.74, 9.42)	349265.81	7.81	1.05 (0.99, 1.10)	0.103		1.06 (1.01, 1.12)	0.032	
D7	30032	2794	9.30 (8.96, 9.65)	348135.40	8.03	1.03 (0.98, 1.09)	0.210		1.05 (1.00, 1.11)	0.060	
D8	30033	3000	9.99 (9.63, 10.35)	346728.19	8.65	1.06 (1.01, 1.12)	0.018		1.09 (1.03, 1.14)	0.002	
D9	30033	3407	11.34 (10.96, 11.73)	343090.84	9.93	1.13 (1.07, 1.19)	<.001		1.15 (1.10, 1.21)	<.001	
D10	30032	3554	11.83 (11.44, 12.22)	339683.25	10.46	1.06 (1.00, 1.11)	0.037		1.09 (1.04, 1.15)	0.001	

Multivariable model (1) was adjusted for age, sex, body mass index, income levels, smoking, alcohol consumption, regular physical activity, hypertension, diabetes mellitus, dyslipidemia, stroke, atrial fibrillation, renal disease, cancer, and on lipid-lowering agent.

Multivariable model (2) was adjusted for age, sex, body mass index, income levels, smoking, alcohol consumption, regular physical activity, hypertension, diabetes mellitus, dyslipidemia, stroke, atrial fibrillation, renal disease, cancer, on lipid-lowering agent, and mean TC.

HR, hazard ratio; CI, confidence interval; CV, coefficient of variation; D, decile; SD, standard deviation; VIM, variability independent of the mean; TC, total cholesterol variability.

Table S8. The risk for the occurrence of vertebral fractures according to the quartiles of total cholesterol variability.

	Number of participants	Number of events	Event rate (%) (95% CI)	Person-years	Incidence rate (per 1000 person-years)	Adjusted HR (95% CI)	*P*-value	*P*-value for trend
CV								<.001
Q1	75081	3690	4.91 (4.76, 5.07)	891640.50	4.14	1 (reference)		
Q2	75082	3534	4.71 (4.55, 4.86)	892073.54	3.96	1.01 (0.96, 1.05)	0.786	
Q3	75082	3853	5.13 (4.97, 5.29)	887983.03	4.34	1.06 (1.01, 1.11)	0.013	
Q4	75081	4843	6.45 (6.27, 6.63)	872670.47	5.55	1.12 (1.07, 1.17)	<.001	
SD								<.001
Q1	75095	3764	5.01 (4.85, 5.17)	889917.37	4.23	1 (reference)		
Q2	75062	3490	4.65 (4.50, 4.80)	891266.56	3.92	0.96 (0.92, 1.01)	0.113	
Q3	75087	3816	5.08 (4.92, 5.24)	888153.34	4.30	1.00 (0.96, 1.05)	0.863	
Q4	75082	4850	6.46 (6.28, 6.64)	875030.27	5.54	1.07 (1.02, 1.12)	0.004	
VIM								<.001
Q1	75081	3764	5.01 (4.85, 5.17)	889755.35	4.23	1 (reference)		
Q2	75082	3490	4.65 (4.49, 4.80)	891504.04	3.91	0.96 (0.92, 1.01)	0.109	
Q3	75082	3816	5.08 (4.92, 5.24)	888083.22	4.30	1.00 (0.96, 1.05)	0.868	
Q4	75081	4850	6.46 (6.28, 6.64)	875024.92	5.54	1.07 (1.02, 1.12)	0.004	

Multivariable model was adjusted for age, sex, body mass index, income levels, smoking, alcohol consumption, regular physical activity, hypertension, diabetes mellitus, dyslipidemia, stroke, atrial fibrillation, renal disease, cancer, on lipid-lowering agent, and mean TC.
HR, hazard ratio; CI, confidence interval; CV, coefficient of variation; Q, quartile; SD, standard deviation; VIM, variability independent of the mean.

Table S9. The risk for the occurrence of hip fractures according to the quartiles of total cholesterol variability.

	Number of participants	Number of events	Event rate (%) (95% CI)	Person-years	Incidence rate (per 1000 person-years)	Adjusted HR (95% CI)	*P*-value	*P*-value for trend
CV								<.001
Q1	75081	562	0.75 (0.69, 0.81)	906053.50	0.62	1 (reference)		
Q2	75082	536	0.71 (0.65, 0.77)	906273.08	0.59	1.04 (0.93, 1.17)	0.506	
Q3	75082	629	0.84 (0.77, 0.90)	902787.68	0.70	1.18 (1.05, 1.32)	0.005	
Q4	75081	945	1.26 (1.18, 1.34)	890470.11	1.06	1.37 (1.23, 1.52)	<.001	
SD								0.001
Q1	75095	596	0.79 (0.73, 0.86)	904499.49	0.66	1 (reference)		
Q2	75062	562	0.75 (0.69, 0.81)	904961.52	0.62	1.03 (0.92, 1.16)	0.611	
Q3	75087	626	0.83 (0.77, 0.90)	902889.35	0.69	1.10 (0.98, 1.23)	0.110	
Q4	75082	888	1.18 (1.10, 1.26)	893234.01	0.99	1.22 (1.10, 1.36)	<.001	
VIM								0.001
Q1	75081	596	0.79 (0.73, 0.86)	904337.47	0.66	1 (reference)		
Q2	75082	562	0.75 (0.69, 0.81)	905199.00	0.62	1.03 (0.92, 1.16)	0.617	
Q3	75082	626	0.83 (0.77, 0.90)	902819.23	0.69	1.10 (0.98, 1.23)	0.110	
Q4	75081	888	1.18 (1.10, 1.26)	893228.66	0.99	1.22 (1.10, 1.36)	<.001	

Multivariable model was adjusted for age, sex, body mass index, income levels, smoking, alcohol consumption, regular physical activity, hypertension, diabetes mellitus, dyslipidemia, stroke, atrial fibrillation, renal disease, cancer, on lipid-lowering agent, and mean TC.

HR, hazard ratio; CI, confidence interval; CV, coefficient of variation; Q, quartile; SD, standard deviation; VIM, variability independent of the mean.

Table S10. The risk for the occurrence of distal radius fractures according to the quartiles of total cholesterol variability.

	Number of participants	Number of events	Event rate (%) (95% CI)	Person-years	Incidence rate (per 1000 person-years)	Adjusted HR (95% CI)	*P*-value	*P*-value for trend
CV								0.388
Q1	75081	2696	3.59 (3.46, 3.73)	892361.23	3.02	1 (reference)		
Q2	75082	2692	3.59 (3.45, 3.72)	892725.49	3.02	1.01 (0.96, 1.07)	0.605	
Q3	75082	2843	3.79 (3.65, 3.93)	888430.23	3.20	1.02 (0.97, 1.08)	0.446	
Q4	75081	3244	4.32 (4.17, 4.47)	874893.19	3.71	1.05 (0.99, 1.10)	0.091	
SD								0.199
Q1	75095	2696	3.59 (3.45, 3.73)	890990.79	3.03	1 (reference)		
Q2	75062	2591	3.45 (3.32, 3.58)	892231.85	2.90	0.96 (0.91, 1.02)	0.165	
Q3	75087	2888	3.85 (3.71, 3.99)	888227.15	3.25	1.01 (0.96, 1.06)	0.804	
Q4	75082	3300	4.40 (4.25, 4.55)	876960.35	3.76	1.02 (0.97, 1.07)	0.518	
VIM								0.195
Q1	75081	2696	3.59 (3.46, 3.73)	890828.77	3.03	1 (reference)		
Q2	75082	2591	3.45 (3.32, 3.58)	892469.33	2.90	0.96 (0.91, 1.02)	0.161	
Q3	75082	2888	3.85 (3.71, 3.99)	888157.03	3.25	1.01 (0.96, 1.06)	0.809	
Q4	75081	3300	4.40 (4.25, 4.55)	876955.01	3.76	1.02 (0.97, 1.07)	0.522	

Multivariable model was adjusted for age, sex, body mass index, income levels, smoking, alcohol consumption, regular physical activity, hypertension, diabetes mellitus, dyslipidemia, stroke, atrial fibrillation, renal disease, cancer, on lipid-lowering agent, and mean TC.
HR, hazard ratio; CI, confidence interval; CV, coefficient of variation; Q, quartile; SD, standard deviation; VIM, variability independent of the mean.

Table S11. The risk for the occurrence of humerus fractures according to the quartiles of total cholesterol variability.

	Number of participants	Number of events	Event rate (%) (95% CI)	Person-years	Incidence rate (per 1000 person-years)	Adjusted HR (95% CI)	*P*-value	*P*-value for trend
CV								0.591
Q1	75081	397	0.53 (0.48, 0.58)	906182.63	0.44	1 (reference)		
Q2	75082	401	0.53 (0.48, 0.59)	906344.47	0.44	1.04 (0.91, 1.20)	0.565	
Q3	75082	391	0.52 (0.47, 0.57)	903224.00	0.43	0.98 (0.85, 1.12)	0.738	
Q4	75081	496	0.66 (0.60, 0.72)	891178.89	0.56	1.06 (0.93, 1.22)	0.367	
SD								0.639
Q1	75095	378	0.50 (0.45, 0.55)	904784.40	0.42	1 (reference)		
Q2	75062	405	0.54 (0.49, 0.59)	905222.57	0.45	1.09 (0.95, 1.26)	0.225	
Q3	75087	407	0.54 (0.49, 0.59)	903097.78	0.45	1.04 (0.90, 1.19)	0.616	
Q4	75082	495	0.66 (0.60, 0.72)	893825.24	0.55	1.07 (0.93, 1.23)	0.347	
VIM								0.642
Q1	75081	378	0.50 (0.45, 0.55)	904622.39	0.42	1 (reference)		
Q2	75082	405	0.54 (0.49, 0.59)	905460.05	0.45	1.09 (0.95, 1.26)	0.227	
Q3	75082	407	0.54 (0.49, 0.59)	903027.66	0.45	1.04 (0.90, 1.19)	0.617	
Q4	75081	495	0.66 (0.60, 0.72)	893819.90	0.55	1.07 (0.93, 1.23)	0.348	

Multivariable model was adjusted for age, sex, body mass index, income levels, smoking, alcohol consumption, regular physical activity, hypertension, diabetes mellitus, dyslipidemia, stroke, atrial fibrillation, renal disease, cancer, on lipid-lowering agent, and mean TC.
HR, hazard ratio; CI, confidence interval; CV, coefficient of variation; Q, quartile; SD, standard deviation; VIM, variability independent of the mean.
