# Supplementary figures and images for: Total Cholesterol Variability and the Risk of Osteoporotic Fractures: A Nationwide Population-Based Cohort Study
Source: J Pers Med. 2023 Mar 11;13(3):509. doi: 10.3390/jpm13030509 (PMC10054569; doi:10.3390/jpm13030509)

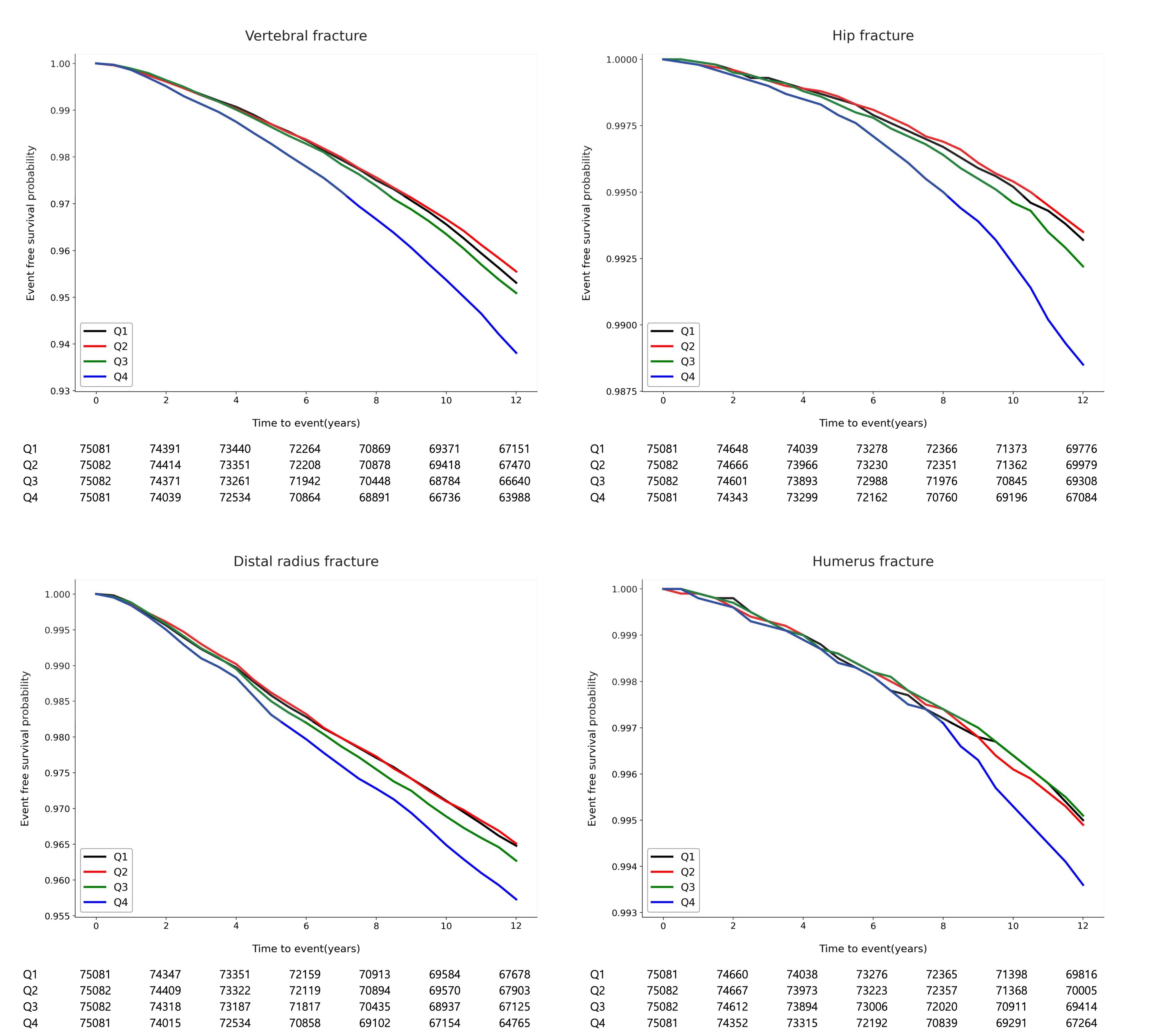

Supplement: Supplementary file 1 [file jpm-13-00509-s001.zip › 2022-11-14 TC-osteoporotic_Fx_Supplementary Figure S1. TC_OF KMplot.jpg]
